# Effects of immersive virtual reality intervention on pain and anxiety among pediatric patients undergoing venipuncture: a study protocol for a randomized controlled trial

**DOI:** 10.1186/s13063-019-3443-z

**Published:** 2019-06-20

**Authors:** Cho Lee Wong, Miranda Mei Wa Lui, Kai Chow Choi

**Affiliations:** 1 The Nethersole School of Nursing, Faculty of Medicine, The Chinese University of Hong Kong, Room 824, 8/F, Esther Lee Building, The Chinese University of Hong Kong, Shatin, Hong Kong, People’s Republic of China; 2 Paediatrics & Adolescent Medicine, Tseung Kwan O Hospital, Hospital Authority, Hong Kong, People’s Republic of China

**Keywords:** Anxiety, Immersive virtual reality, Pain, Pediatric patients

## Abstract

**Background:**

Venipuncture is one of the most painful and distressing procedure experienced by pediatric patients. Evidence suggests that distraction combined with age-appropriate procedural information can effectively decrease procedural pain and anxiety in pediatric patients. Immersive virtual reality (IVR) can simultaneously provide complete distraction and procedural information to patients.

**Methods:**

Guided by the gate control theory and Lazarus and Folkman’s theory, this study aims to examine the effects of IVR intervention on reducing the pain, anxiety and stress, the duration of venipuncture, and the satisfaction of healthcare providers for the procedure. A randomized controlled trial with repeated assessments will be conducted. A total of 200 pediatric patients aged 4–12 years will be recruited from a regional public hospital and randomly assigned to either the intervention or control group. The study will use two age-appropriate IVR modules that consist of procedural information. The intervention group will receive IVR intervention, whereas the control group will receive standard care only. The cost-effectiveness of IVR intervention will be compared with that of standard care. Outcome evaluation will be conducted at four time points: 10 min before, during, immediately after, and 30 min after the procedure. Intention to treat and generalized estimating equation model will be used to analyze the data.

**Discussion:**

This study is the first of its kind to adopt IVR intervention with age-appropriate procedural information for pediatric patients undergoing venipuncture. Findings of the proposed study may: (1) provide a novel, facile, and cost-effective intervention that can be used virtually at any time and place to manage pain and anxiety; and (2) shed light on the global trends of research and clinical development of IVR as an intervention for other painful and stressful medical procedures.

**Trial registration:**

Chinese Clinical Trial Registry, ChiCTR1800018817. Registered on 11 October 2018.

**Electronic supplementary material:**

The online version of this article (10.1186/s13063-019-3443-z) contains supplementary material, which is available to authorized users.

## Background

Hospitalized children experience pain and anxiety from invasive procedures and/or their underlying diseases [1]. Venipuncture, a frequently performed needle-related procedure, is one of the most frightening experiences and a common source of moderate to severe pain for pediatric patients [1, 2]. The pain associated with the procedure is secondary only to the illness itself [3]. Approximately 83% of children aged 2.5–6 years, 51% of children aged 7–12 years, and 28% of adolescents (aged > 12 years) who underwent venipuncture reported high levels of distress during the procedure [4]. However, < 10% of venipuncture procedures are provided with pain management [2].

Inadequate pain management can negatively affect children, parents, and medical institutions [5–10]. Unmanaged needle-related procedural pain in children is associated with increased pain and stress during subsequent procedures, fear and avoidance of medical care, or even the development of needle phobias that persist into adulthood [3, 6–8]. Failure to manage needle-related pain may increase the manpower, resources, and time required to complete procedures, consequently reducing healthcare providers’ satisfaction with respect to the procedures [5, 10].

Pre-procedural local analgesics have been traditionally used to manage needle-related procedural pain. However, high levels of pain and distress were reported in children even with pharmacological interventions [11, 12]. Thus, clinical guidelines highlight the important role of non-pharmacological interventions alone or as clinical adjunct in managing procedural pain [13].

### Non-pharmacological interventions

Various studies have suggested non-pharmacological interventions, such as distraction, procedural information provision, hypnosis, and cognitive behavioral therapy, to manage the pain and anxiety experienced by pediatric patients undergoing needle-related procedures [14–16]. However, distraction and information provision are more feasible to implement than previously suggested interventions because many clinicians are not trained in hypnosis or cognitive behavioral therapy.

Distraction is the most effective non-pharmacological intervention for mitigating the pain and anxiety experienced by pediatric patient undergoing needle-related procedures [14–16], particularly in children aged < 12 years [16]. A review of 26 studies involving 2548 children aged 2–19 years showed that needle-related procedures have been performed with various distractors, such as listening to music, watching cartoons, playing with toys, and mother-directed distractions, such as soothing [14]. The review suggested that distractions without adult involvement (supportive role articulated for the adult within the intervention) and child choice effectively reduce the intensity of self-reported distress [14]. Two recent studies further confirmed that distraction intervention integrated with procedural information produces significant outcomes [17, 18]. However, simultaneously providing age-appropriate distraction and procedural information may be difficult and may likely increase the workload of hospital staff. Most importantly, previously employed distractors (e.g. music, cartoon, or toys) failed to provide complete distraction because children could still see the needles, which is the most distress-evoking experience in needle-related procedures [5, 6]. Thus, an intervention that can provide age-appropriate procedural information and completely distract the attention of children from painful stimuli should be identified.

### Immersive virtual reality

Immersive virtual reality (IVR) may help overcome the above obstacles. It provides a means of human/computer interaction, wherein a human becomes an active participant in a virtual environment created through a head-mounted display [19]. The user is immersed and actively participates in the virtual environment as it changes in real time with the user’s movements [20]. Moreover, it can be used at any time and place in clinical settings without requiring extra manpower. IVR modules can also be tailored to provide health information to the user. The cost of IVR equipment has also become increasingly affordable. Distraction-based IVR intervention has already been implemented in the Lucile Packard Children’s Hospital at Stanford in the United States [21].

### Studies of the effects of IVR on procedural pain and anxiety

A large body of evidence supports the efficacy of IVR in reducing pain, anxiety, and stress among pediatric patients undergoing burn care or cancer treatments [19, 20]. With regard to needle-related procedures, a randomized control trial (RCT) was conducted with 20 pediatric patients aged 8–12 years and who required peripheral intravenous access [11]. The intervention group received IVR for 5 min before the procedure, whereas the control group received local anesthetic spray without IVR. Pain was measured using the Faces Pain Scale-Revised (FPS-R). Participants in the intervention group reported that their perceived pain was not significantly different, whereas those in the control group reported a fourfold increase in pain following the procedure [11]. Two studies involved pediatric oncology patients aged 7–19 years and who were undergoing port access (insertion of a needle into an implanted device to facilitate blood collection or injections). Participants in the intervention group were immersed in a virtual gorilla habitat. Results found that the heart rates of participants in the intervention group significantly decreased, indicating that children in the intervention group did not experience as much pain and anxiety as those in the control group [22, 23]. Another study on children with cancer (aged 5–18 years) undergoing venipuncture (*n* = 4) or port access (*n* = 46) allowed patients in the intervention group to select from various immersive and non-immersive distractors (such as books, IVR, music, and video games) [24]. The self-reported pain of patients in the IVR group was no different. However, a reduction of fear and distress was reported by the outcome assessors in IVR group.

Although previous studies have provided some positive findings regarding the effects of IVR on needle-related procedural pain and anxiety, these studies involved small sample sizes (*n* = 20 to 59) [11, 22–24] and recruited children of various developmental ages (5–19 years) without using age-appropriate IVR modules [11, 22–24]. Most importantly, all these studies adopted IVR as a distraction intervention only. To our knowledge, no study has utilized IVR to provide distraction and procedural information to patients undergoing venipuncture. Given that venipuncture is the most painful and fearful procedure for pediatric patients [1–4], future large-scale studies on the efficacy of IVR to provide complete distraction and procedural information to pediatric patients undergoing venipuncture may offer crucial insight on the application of this approach.

### Theoretical frameworks

The gate control theory and Lazarus and Folkman’s theory provide the theoretical underpinning of this study [25, 26]. The gate control theory suggests that peripheral nerves become excited when cells are damaged. Impulses from nerves pass along to spinal cord systems and other neuroanatomical structures before reaching the cerebral cortex where pain is perceived. The gate control system in the spinal cord opens and closes to modulate pain perception. If non-nociceptive input (IVR) exceeds the nociceptive (pain) input, then the gate can partially or entirely close, blocking the transmission of the pain signal to the brain. The theory also proposes that pain signals that descend from the brain through the gate can be amplified by emotional experiences, such as anxiety and stress. Lazarus and Folkman’s theory [26] states that an individual’s evaluations of anxiety and stress-provoking experiences are influenced by their perceptions of control over a potential threat. Providing information may help improve pediatric patients’ sense of control over a procedure [17, 18, 27].

In the context of these two theories, IVR functions by providing multisensory input to divert the children’s conscious attention from venipuncture-associated pain, thus helping close gate control and decreasing pain perception [28]. The analgesic effects of IVR have been supported by the results of functional magnetic resonance imaging assessment [29].

## Objectives

1. To compare the effectiveness of IVR intervention with standard care in pediatric patients undergoing venipuncture on pain, anxiety, stress, and length of procedure.
2. To compare the satisfaction of healthcare providers that use IVR intervention and standard care.
3. To evaluate cost-effectiveness of IVR intervention compared to standard care for pediatric patients undergoing venipuncture.

### Hypotheses

1. Compared with standard care, IVR intervention significantly reduces pain, anxiety, stress, and length of procedure in pediatric patients undergoing venipuncture.
2. Compared with standard care, IVR intervention significantly improves the satisfaction of healthcare providers toward the venipuncture procedures.
3. IVR intervention is significantly more cost-effective than standard care for pediatric patients undergoing venipuncture.

## Methods

### Design

This is a two-arm parallel RCT.

### Study setting

This study will be conducted in the pediatric unit of a public hospital. The unit admit general pediatric patients. Venipuncture will be performed by a doctor or trained phlebotomist and will be conducted in the treatment room. No local analgesics will be applied before the procedure. Usually at least one staff (nurse or healthcare assistant) will assist in the procedure (by saying comfort words and restraining the child from vigorous movement). Parents will wait outside the treatment room during the procedures.

### Participants

Eligible pediatric patients: (1) aged 4–12 years; (2) scheduled to undergo venipuncture; and (3) can understand Chinese and follow instructions. Potential participants will be excluded according to the following: (1) identified cognitive and learning problems in their medical record; (2) sensory impairment to pain (such as spinal bifida); (3) identified contact precautions; and (4) previous history of seizures or motion sickness. This study selects 4–12-year-old patients because they experience high level of distress in venipuncture procedure (4) and distraction is efficacious in this age group [14]. Younger patients will not be included because of their possible limited cognitive and verbal capacity to respond to the questionnaires.

### Sample size: patients

In 2016, about 250 hospitalized patients aged 4–12 years were admitted to the unit and required venipuncture. Sample size estimation was based on the effect estimated from a previous IVR study using the FPS-R Scale as a primary outcome measure [11]. By using the power analysis software, GPower 3.1, it was estimated that a sample size of 85 participants per group would enable a two-arm RCT to detect a between-group difference of 0.8 in FPS-R scale with a pooled standard deviation of 1.84 with 80% power at 5% level of significance. Taking into account an attrition rate of up to 15% [23], 200 children will be recruited for the experimental and control groups, with 100 children in each group.

#### Healthcare providers

All healthcare providers involved in venipuncture procedures (e.g. doctors, phlebotomist, and healthcare assistants) will be invited to assess their satisfaction for the procedures.

### Randomization

Eligible participants will be randomly assigned in a 1:1 ratio to the intervention group who will receive the IVR intervention or a control group that will receive standard care only using stratified permuted block randomization with a block size of 10 to maintain a good balance of participants between the two groups throughout the participant recruitment period. Randomization will be stratified by age group (4–7 years, 8–12 years) in equal numbers. According to Piaget’s theory, children aged 4–7 years belong to the same pre-operational stage, whereas those aged 8–12 years belong to the concrete operational stage [30]. Children in different stages differently perceive information and pain stimuli sensitivity [31].

A sequence of grouping identifiers (I = intervention group or C = control group) will be prepared in advance by an independent statistician, using computer-generated random codes for each of the two strata of age group. The group identifiers for each age group will then be put in serially numbered sealed opaque envelopes according to the underlying random sequence list by the statistician. The group allocation of the patients will be assigned according their ages, sequence of enrolment in the study, and the group identifier contained in the corresponding numbered envelopes. Group allocation will be concealed from the research assistant (RA), ward staff, child, and parent until consent and baseline assessment data have been obtained. Blinding of participants will be difficult but will not necessarily contribute to a source of bias because children are unlikely to change their behavior even when they know they are participating in a certain intervention [32].

### Intervention: IVR

In addition to standard care, children in the intervention group will receive IVR intervention through a commercially available disposable headset (Google cardboard goggle), which can be fitted into majority of commonly available smartphones. Although other latest headsets, such as Oculus Rift or HTC Vive, can provide a high-quality immersion experience, they likewise increase the risk of contact infection and require a high spectrum personal computer to operate. Therefore, these devices will not be used in this study.

With regard to the IVR modules, pilot work conducted by our research team found that cartoon animation was preferred by young patients, whereas the interactive game was preferred by adolescents; these results concurred with a previous study [24]. An IVR module that presents procedural information through a customized and child-friendly design more effectively mitigates pain and distress than that with off-the-shelf content [17, 29, 33]. However, these freely downloaded IVR modules that were developed in Western countries with English as the medium may not be completely appropriate for pediatric patients in Hong Kong. In the proposed study, an IVR module will be designed on the basis of suggestions to use age-appropriate modules for patients [16, 34] and the experiences of the PI, who has successfully developed six cartoon animations in previous studies [35]. The PI produced two developmentally appropriate animation modules: one for children aged 4–7 years and one for children aged 8–12 years; module content were validated by an expert panel (consists of pediatricians, nurses, and professors) and children. The two modules share the following common characteristics with those used in previous studies: (1) a wide range of visual and auditory stimuli [11, 22, 23]; (2) requiring minimum movement of head and hand to allow the procedures to proceed unhindered [36]; and (3) provision of procedural information. Each module is about 10 min in duration.

### Virtual reality storyline

The animation follows the story of a young character called “DD,” who resides in outer space and is a fellow patient at the hospital. Like the patient, DD is also about to receive the venipuncture treatment and she wants to support other pediatric patients in the treatment. DD mentions her satisfaction and comfort with the hospital staff environment before introducing the procedures of the venipuncture, which is described as an adventurous mission. DD reminds the patient to remain calm and to be courageous in order to complete the mission and also describes certain sensations that the patient may feel. After the procedure is over, DD congratulates and awards the patient, and then guides the patient around outer space.

#### Module for children aged 4–7 years

For children aged 4–7 years, their comprehension of words and sentences is not well-established. However, their curiosity and imaginative thinking are developed [37]. On the contrary, visual stimulation may be effective in distracting the children [27, 38]. Thus, this animation presents fast-paced zooming of the screen with cartoon characters enacting various body movements [38]. Pastel tone colors which are less tiring to the eyes are used to provide visual stimulation. The animation “DD is in the hospital” uses simple words and sentences to provide procedural information to the pediatric patients as to why the cartoon character “DD” needs a venipuncture in a child-focused manner. The animation aims to provide distraction as well as instill a sense of control by exposing patients to the procedure [7, 27, 29].

#### Module for children aged 8–12 years

The main goal of this module is to create an interactive environment for distracting children aged 8–12 years during venipuncture. The animation will prepare children for venipuncture by providing information, such as: (1) why the procedure must be done; (2) what will happen; and (3) how the procedure will feel. The animation will be followed by an interactive game to increase patients’ sense of control over the procedure. In this game, the patients will help to perform venipuncture on a cartoon character in the IVR environment [27, 38].

### Implementation protocol

The venipuncture procedure will be conducted in the treatment room while parents wait outside. Patients assigned to the intervention group will receive IVR intervention 5 min before the start of venipuncture until the end of the procedure [11, 22]. The RA will provide simple and standard instruction on how to use the equipment. The headset will then be placed on patient’s head and adjusted to ensure a comfortable and secure fit. Patients will be allowed to view the IVR module according to their age group (4–7 years, 8–12 years). During the intervention, the head movement of the patient will control the IVR module. The patients will also be told that the intervention will be discontinued if they experience motion sickness, eye discomfort, or headaches.

After receiving IVR intervention for 5 min, the RA will invite the doctor/phlebotomist to start the venipuncture. The beginning of the procedure is indicated by doctor/phlebotomist disinfecting the venipuncture site. The RA will note the time of first attempt at venipuncture. The end of the procedure is indicated by application of band aid to the venipuncture site. The RA will remove the IVR equipment from the child after the procedure. The protocol follows for the first venipuncture attempt only. If the first attempt is unsuccessful, additional attempts occurring after the protocol will be completed, but participants will be withdrawn from study.

### Fidelity of the intervention

The fidelity of the intervention will be ensured by recruiting a RA with a minimum of two years of experiences in pediatric care. She will undergo two days of training conducted by the PI. The training will include: (1) basic knowledge about and application of IVR; (2) procedures of implementing the intervention and collecting data; and (3) management of untoward reactions, such as motion sickness. A minimum of one session conducted by the RA each month will be randomly selected to assess compliance with the implementation protocol by the PI. The PI and RA will meet monthly to discuss the delivery of IVR intervention and study progress. Feedback will be given accordingly.

### Control group: standard care

Participants in the control group will receive standard care without IVR intervention. Standard care includes explaining why and what is being done and saying comforting and supportive words during procedures.

### Outcome measures

#### Primary outcome

##### Faces pain scale-revised

The FPS-R scale is a scale of 0–10 comprising six horizontally arranged cartoon faces with expressions of “0 = no pain” to “10 = very painful” [39]. Participants will be asked to point to the face that indicates how much pain she/he feels. The protocol for use of FPS-R is standardized and is a reliable and valid scale for evaluating pain in children [9, 11].

#### Secondary outcomes

##### Visual analogue scale for anxiety

A visual analogue scale (VAS) for anxiety will be used to assess the anxiety levels of children aged 4–7 years. The VAS is a 10-cm horizontal line marked with the words “not worried” (low score) at one end and “very worried” (high score) at the other, with different facial expressions drawn along the line. Children aged 4–7 years will be asked to indicate their levels of anxiety by moving a pointer over the line, with higher scores indicating greater anxiety. The VAS is a widely used scale which is reliable and valid for measuring the subjective feelings of children [40]. It has been used previously by PI to assess the anxiety level of children undergoing medical procedures [41].

##### State anxiety scale for children

The short form of the Chinese version of the State Anxiety Scale for Children (CSAS-C) will be used to measure the anxiety levels of children aged 8–12 years [42]. The CSAS-C is a 3-point Likert scale with total scores in the range of 10–30. Higher scores indicate greater anxiety levels [41]. The psychometric properties of the short form have been tested and found to correlate strongly with the full form (r = 0.92). It has good internal consistency (r = 0.83) and convergent validity that differentiate the anxiety state of children under various situations [43]. The PI has previously used it to assess the anxiety level of children undergoing medical procedures with Cronbach’s alpha 0.80–0.88 [41].

##### Heart rate

The heart rate of the children will be measured by a standard automatic heart rate monitoring machine (available in the study institution) to assess the physiological responses of children. Heart rate is considered to be objective and definitive in indirectly assessing physiological responses of pain and anxiety of children [22, 23].

##### Salivary cortisol assay

Saliva cortisol assay will be used as to assess the stress levels of the children [44]. The trained RA will collect saliva samples from the patients using the Salivette sampling devices, according to the manufacturer’s instructions at 10 min before and 30 min after the venipuncture procedures. Patients will be advised not to brush their teeth or eat 2 h before saliva sample collection to avoid contamination because acidic or high-sugar foods can compromise essay performance by lowering sample pH and influencing bacterial growth [44]. Collected saliva samples will be frozen at – 80 °C until further processing and analysis. Cortisol levels in the saliva samples will be measured using an enzyme-linked immunoassay kit (Salimetrics, PA, USA), according to the manufacturer’s instructions.

##### Length of procedure

A standard stopwatch will be used to measure the length of the procedure from the beginning (time when the doctor/phlebotomist starts to disinfect the site) to the end of procedure (time when applies the band aid on the venipuncture site).

##### Staff satisfaction scale

The staff satisfaction scale will be adopted to measure the satisfaction levels of the healthcare providers toward the procedure [45]. It consists of eight items and each is rated by a 5-point scale ranging from 1 = strongly disagree to 5 = strongly agree. Higher score presents a higher level of the satisfaction. This scale has been translated by the PI using back-translation method recommended by Brislin (1970) and used in a previous study with the Cronbach’s alpha of 0.90 [41].

##### Cost-effectiveness

Cost analysis will be examined based on incremental cost-effectiveness ratio expressed as incremental cost per every unit decrease in the primary outcome of the FPS-R scale immediately after intervention with respect to the baseline. All the cost data involved will be expressed in Hong Kong dollars and valued on the starting date of the study on the basis of non-subsidized cost. All costs incurred will be estimated per each participant using the method of Thompson and Barber [46]. Specifically, the cost of human resources (e.g. doctors, nurses, phlebotomist, and healthcare assistants) is measured by the salary of the middle rank of the professionals and calculated based on the minutes used to perform the procedures. Costs of consumables to perform the venipuncture (e.g. alcohol pad, glove, butterfly needles, and band aid) will be based on the retail price paid by the finance office of the hospital. In addition, the cost for development of IVR modules and VR cardboard goggles will be included in the intervention group.

##### Data collection procedures

Children requiring venipuncture will be identified by the nurse in the pediatric unit. If the children meet the inclusion criteria for recruitment, the nurse will refer the children and their accompanying parents to the RA in the treatment room, who will give them an information sheet, explain the study, and show the IVR equipment. If they agree to participate, written informed consent from the accompany parent and assent from the children will be obtained. The RA will then acquire socio-demographics information from the parents and clinical characteristics of the children from medical record before randomization. According to the participant allocation scheme, children in the control group will receive standard care, whereas those in the intervention group will also receive IVR intervention.

**Fig. 1:**
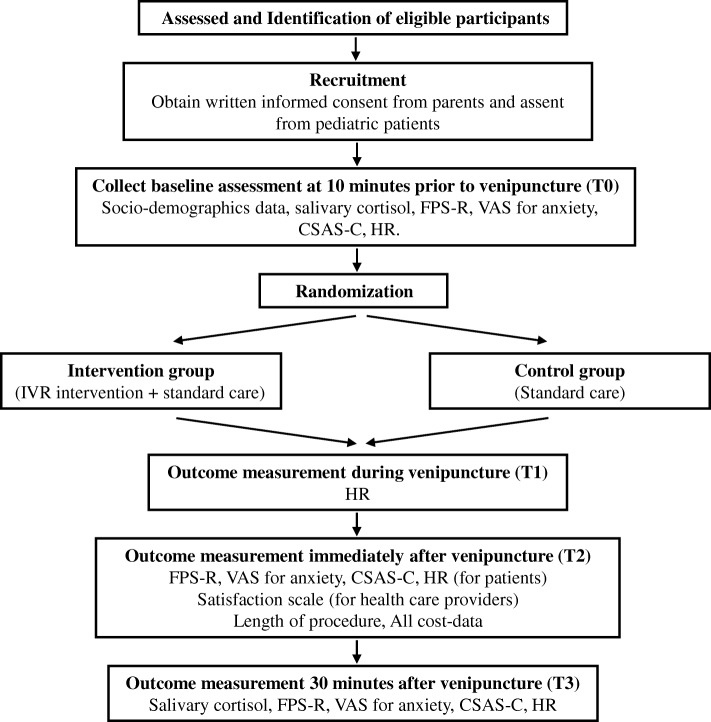
The study *flowchart*. FPS-R Faces Pain Scale-Revised, VAS Visual Analogue Scale, CSAS-C short form Chinese version of the State Anxiety Scale for Children, HR heart rate, IVR immersive virtual reality

Participants will be assessed:10 min before the procedure (T0); during the procedure when the needle is inserted into the skin (T1); immediately after the procedure, indicated by the application of a band aid to the venipuncture site (T2); and 30 min after the procedure (T3). At T0, a set of baseline data – saliva sample for a measure of cortisol, FPS-R, VAS for anxiety, CSAS-C, and heart rate – will be collected from the patients by the RA. At T1, heart rate will be obtained. At T2, FPS-R, VAS for anxiety, CSAS-C, and heart rate will be obtained from children. At the same time, the RA will record the length of procedure and invite the healthcare providers involved to fill in the staff satisfaction scale. At T3, saliva sample for a measure of cortisol, FPS-R, VAS for anxiety, CSAS-C, and heart rate will be obtained from patients again. Please refer to Table 1 and Fig. 1.

**Table 1:** The data collection plan

	10 min before the venipuncture procedure (T0)	During venipuncture procedure (T1)	Immediately after venipuncture procedure (T2)	30 min after venipuncture procedure (T3)
Sociodemographic data	X			
Salivary cortisol	X			X
FPS-R	X		X	X
VAS for anxiety (for children aged 4–7 years)	X		X	X
CSAS-C (for children aged 8–12 years)	X		X	X
HR	X	X	X	X
Staff Satisfaction Scale			X	
The length of procedure			X	
All cost-data			X	

*RPS-R* Faces Pain Scale-Revised, *VAS* visual analogue scale, *CSAS-C* short form of the Chinese version of the State Anxiety Scale for Children, *HR* heart rate

### Ethical consideration

Ethical approval will be sought from the Ethical Committees of the study institutions. Written informed consent from parents, healthcare providers involved in venipuncture procedures, and assent from child will be obtained. Participants and parents will be informed that the care will not be affected by their participation status.

### Data analysis

IBM SPSS 24 will be used for data analysis. Continuous demographic and clinical variables will be presented by their means and standard deviations, whereas categorical data (e.g. sex) will be presented in frequencies and percentages, as appropriate. The intention-to-treat principle will be adopted for the outcome comparisons between the intervention and control arms. Since the anxiety levels of the two age groups will be assessed by two different instruments, the anxiety scores assessed by each instrument will therefore be converted to z scores before pooling for outcome analysis. The generalized estimating equations (GEE) model will be used to compare each of the outcome measures, except the staff satisfaction, across the time points between the two study arms with the dichotomous age group variable set as a covariate to make adjustment for the stratified randomization by age group. GEE model can account for intra-correlated repeated measures data and produce unbiased estimates even if there are missing data, provided that the data are missing at completely random. For the staff satisfaction outcome, depending on the number of staff involved in satisfaction ratings, a mixed-effects model or simply a linear regression model will be used to compare the outcome between the two arms with adjustment for raters’ effects. As the means and standard deviations involved in the z scores conversion of the anxiety scale scores are based on sample’s instead of population’s means and standards, the calculated z scores may not truly reflect the deviations from population mean in units of population standard deviation. Sensitivity analysis will be conducted to examine if the intervention effects on the anxiety level in the two age groups are consistent with the pooled age group. Cohen’s d values will also be calculated to estimate the effect sizes of the IVR intervention on the outcome variables. All statistical analyses are two-sided and level of significance will be set at 0.05.

## Discussion

Venipuncture is the most painful and distressing needle-related procedure experienced by pediatric patients. However, procedural pain remains underdiagnosed and undertreated in this vulnerable group. Given the negative consequences of unmanaged procedural pain to patients, parents, and medical institutions, effective intervention is needed to minimize pain and distress experienced by pediatric patients undergoing venipuncture. Providing interventions that integrate distraction with age-appropriate procedural information can effectively mitigate pain and anxiety among pediatric patients undergoing venipuncture. However, previously adopted distractors did not provide complete distraction. Furthermore, simultaneously providing distraction with procedural information is difficult and requires additional manpower and time.

IVR can fully engage patients in an immersive environment and distract their attention from noxious stimuli while simultaneously providing procedural information. Guided by the gate control theory and the Lazarus and Folkman’s theory, this study aims to examine the effects of IVR intervention on reducing pain, anxiety, stress, and length of procedure among pediatric patients undergoing venipuncture. The satisfaction ratings of healthcare providers for the procedures and cost-effectiveness of IVR will also be evaluated.

The findings of this study will likely generate a long-term impact. First, it will help to advance the understanding of gate control theory and the Lazarus and Folkman’s theory. Guided by these two theories, this study is the first of its kind to adopt a robust design and a large sample to examine the effectiveness of IVR in distracting and providing procedural information to pediatric patients undergoing venipuncture. Results of this study not only provide insights for intervention mechanisms, it will also provide implications for theories development or extension.

Second, this study may have translational potential and significantly affect society. If proven effective, IVR can be adopted as a high-quality clinical intervention for mitigating pain and anxiety among pediatric patients undergoing venipuncture. The principles of this intervention can be generalized and extended beyond venipuncture, the ultimate impact is to benefit not only pediatric patients undergoing venipuncture, but also other needle-related or medical procedures that are painful and anxiety-inducing. The integration of the research findings into clinical practice will also enhance the knowledge of healthcare providers and keep them updated on the changes and translation of evidence-based knowledge into clinical practice.

Third, this study will likely generate positive findings and thus contribute tangible improvements to patient care and outcomes. The IVR intervention will not only reduce the pain, anxiety, and stress experienced by patients during venipuncture but also decrease the duration of the procedure. Furthermore, it may also reduce the use of restraints during venipuncture because it is expected to increase the cooperativeness of patients. Pain and restraint reduction are important quality benchmarks in child health delivery systems. Therefore, the quality of patient care and health outcomes can be improved through the implementation of IVR. Moreover, the satisfaction of healthcare providers toward the procedures will likely increase Additional file 1.

### Summary

The proposed IVR intervention will potentially provide significant practical implications in addressing pain, anxiety, and stress of the patients. The ultimate impact of this intervention is to increase procedural compliance, consequently decreasing the length and cost of the procedure while possibly improving the satisfaction of the healthcare providers with the procedure.

### Trial status

This trial will commence on 1 January 2019. The anticipated end date of the study is 31 December 2020.

## Additional file

Additional file 1: SPIRIT 2013 Checklist: Recommended items to address in a clinical trial protocol and related documents*. (DOC 256 kb)

## Data Availability

The full dataset will be available when it is completed and published. For data requests please contact the Principal Investigator.

## Abbreviations

IVR: Immersive virtual reality
